# Pain, balance, and mobility in people 1 year after total knee arthroplasty: a non-randomized cross-sectional pilot study contrasting posterior-stabilized and medial-pivot designs

**DOI:** 10.1186/s40814-022-01094-0

**Published:** 2022-06-28

**Authors:** Cathy W. T. Lo, Matthew A. Brodie, William W. N. Tsang, Stephen R. Lord, Chun-Hoi Yan, Arnold Y. L. Wong

**Affiliations:** 1 Department of Rehabilitation Sciences, The Hong Kong Polytechnic University, Hong Kong SAR, China; 2 Neuroscience Research Australia, University of New South Wales, Sydney, Australia; 3 Department of Physiotherapy, School of Nursing and Health Studies, Hong Kong Metropolitan University, Hong Kong SAR, China; 4 Department of Orthopaedics and Traumatology, The University of Hong Kong, Hong Kong SAR, China

**Keywords:** Prosthesis, Postural sway, Gait, Wearable sensors, Post-operation, Balance performance

## Abstract

**Background:**

Total knee arthroplasty (TKA) is a common treatment for severe knee osteoarthritis. Medial-pivot TKA systems (MP-TKA) are theoretically better than posterior-stabilized TKA systems (PS-TKA) in improving static and dynamic balance of patients although it is difficult to objectively quantify these balance parameters in a clinical setting.

Therefore, this pilot study aimed to evaluate the feasibility of using wearable devices in a clinical setting to examine whether people with MP-TKA have better postoperative outcomes than PS-TKA, and their balance control is more akin to age-matched asymptomatic controls.

**Methods:**

The current cross-sectional pilot study recruited 57 participants with 2 different prosthesis designs (20 PS-TKA, 18 MP-TKA) and 19 asymptomatic controls. At 1-year post-TKA, pain, knee stiffness, and physical function were assessed using the Western Ontario and McMaster Universities Osteoarthritis Index (WOMAC). Static balance, mobility, and gait stability of the participants were evaluated based on data collected from wearable motion sensors during the near tandem stance, timed-up-and-go, and 6-min walk tests.

**Results:**

Compared to asymptomatic controls, both TKA groups reported significantly more pain and stiffness and demonstrated reduced functional mobility, increased stride-time-variability, and impaired balance. After Bonferroni adjustment, no significant differences in pain, balance, and mobility performance were observed between PS-TKA and MP-TKA participants 1 year after surgery. However, there was a trend for increased anteroposterior sway of the lumbar and head regions in the MP-TKA participants when undertaking the near tandem stance test. The wearable motion sensors were easy to use without any adverse effects.

**Conclusions:**

It is feasible to use wearable motion sensors in a clinical setting to compare balance and mobility performance of patients with different TKA prothesis designs. Since this was a pilot study and no definite conclusions could be drawn, future clinical trials should determine the impacts of different TKA prosthesis designs on post-operative outcomes over a longer follow-up period.

**Supplementary Information:**

The online version contains supplementary material available at 10.1186/s40814-022-01094-0.

## Key messages regarding feasibility

What uncertainties existed regarding the feasibility?

- It was unclear whether wearable motion sensors could be used to evaluate the balance and mobility of patients undergoing two different types of total knee arthroplasty (TKA) prostheses at 1-year follow-up with reference to age-matched asymptomatic controls.
  What are the key feasibility findings?
- Wearable motion sensors were a safe non-invasive tool to evaluate balance and mobility of TKA patients in a clinical setting.
- The results from the wearable motion sensors showed that both TKA groups demonstrated reduced functional mobility, increased stride-time-variability, and poorer balance as compared to age-matched asymptomatic controls.
  What are the implications of the feasibility findings for the design of the main study?
- Wearable motion sensors can be used in a clinical setting to measure balance and mobility of patients following TKA. A randomized controlled trial should be conducted to determine whether the two types of patients display significantly different pain, balance controls, and mobility during the first 2 years after surgery.

## Background

Patients with knee osteoarthritis demonstrate altered gait as indicated by spatio-temporal (e.g., greater strike duration and decreased cadence) and kinematic gait deviations (e.g., reduced knee flexion excursion during loading) from healthy counterparts during level walking [1]. Total knee arthroplasty (TKA) is a common procedure for treating patients with advanced and symptomatic knee osteoarthritis for pain relief and improved physical function [2]. In response, TKA implants with differing “guided motion” systems have been designed to restore natural knee joint kinematics.

The posterior-stabilized (PS) knee system was developed in 1978 to mimic the posterior-cruciate ligament function [3]. Specifically, the PS knee system uses a “cam-and-post” mechanism in which the mechanical role of the posterior-cruciate ligament is replaced by a post on the inlay and a cam on the femoral component [4, 5]. The post and cam interact to prevent the anterior translation of the femur on the tibia while providing medial and lateral femoral posterior translation during knee flexion [4–6]. Reported advantages include good soft tissue balancing, a large range of motion, predictable joint biomechanics, and the prevention of posterior tibial subluxation [7].

The medial-pivot (MP) knee system was developed in the early 1990s based on the concept of medial centered rotation of a ball-and-socket joint as determined from knee kinematic studies [8–10]. The natural knee kinematics in the MP knee system is replicated by using a more conforming surface in the tibial insert [11]. This ultra-congruent medial compartment provides minimal anteroposterior motion, while the lateral compartment allows anteroposterior translation around a medial axis of rotation during knee flexion [11].

Although patients with TKA display deficits in gait (e.g., range of motion or kinetics) and physical function (including balance) as compared to age-matched asymptomatic controls [1, 12], it remains unclear if the deficits are prosthesis-related [13–15]. Without such information, it is difficult for clinicians to make clinical decisions with respect to the appropriate knee implant for their patients. Different TKA designs should theoretically alter knee kinematics and associated balance and mobility performance [13]. A recent in vitro study using ultrasonic 3-dimenstional motion analysis observed significant differences in tibiofemoral kinematics during simulated deep knee flexion from 20**°** to 120**°** of flexion between PS- and MP-TKA implanted human knee specimens [5]. However, Bae et al. found no significant differences in clinical outcomes (including self-reported pain and physical function, knee range of motion, femorotibial angle, patella tilt angle, and the post-operative patellar translation) between the PS- and MP-TKAs at a post-operative 5-year follow-up study [16].

While optoelectronic capturing systems, instrumented treadmills and force platforms are traditionally used for gait analysis following TKA [13, 17, 18], wearable inertial sensors have been increasingly used as an inexpensive means to quantify gait in a non-laboratory environment [19]. Since wearable devices can measure the three-dimensional acceleration of body parts and estimate velocity, displacement, and gait stability and variability [19–21], and can characterize altered pattern in patients with TKA during functional tasks. Data from wearable devices attached to the head and pelvis have been validated with respect to detecting stride-to-stride oscillations during walking [21], for characterizing patients with Parkinson’s disease and older people at risk of falls [20–22].

Given the above, the primary objective of the pilot study was to explore the feasibility of using wearable motion sensors in a clinical setting to compare: (a) static and dynamic balance, gait stability, and physical performance between people who received PS-TKA and MP-TKA 1 year after surgery; and (b) balance and mobility of these patients and age-matched asymptomatic controls. The secondary objective was to examine the necessity of refining the assessment protocols for different outcome measures so that they can be adopted to a future randomized controlled trial (RCT) to evaluate the effects of different prosthesis designs on physical outcomes of patients, which may inform the decision making in future clinical practice/research. This pilot study was considered as a success in regard to feasibility if the following criteria were met:

- More than 90% of all eligible participants could complete the balance and mobility assessments within 60 min.
- Less than 5% of all recruited participants reported adverse effects during data collection using our wearable sensors.

## Methods

Reporting of the current pilot study was guided by the recommendations of the CONSORT extension to pilot and feasibility trials [23]. (Additional file 1).

### Trial design and participants

This was a non-randomized cross-sectional pilot study involving three groups of participants. Patients with PS- and MP-TKA were recruited from the division of Joint Replacement Surgery from a local Hospital. Potential TKA participants were recruited if they met the inclusion criteria: 60 years or older, independent walking for at least 10 min indoors without using a walking aid, no contraindication to exercises. Participants were excluded if they had a diagnosis of neurological or vestibular impairment, uncontrolled cardiopulmonary disorders, severe diabetes mellitus, rheumatic arthritis, a body mass index (BMI) ≥ 40 kg/m^2^, a knee flexion contracture ≥ 10°, or Kellgren and Lawrence grade ≥ 3 knee osteoarthritis on the non-operated knee indicating absence of moderate or severe arthritis, a history of lower extremity fracture or surgery other than the primary unilateral TKA, and recent lower extremity musculoskeletal injuries that precluded an individual from participating in the study. These patients were excluded because their medical conditions might affect physical performance and confound our findings. Furthermore, patients were excluded if they had major post-operative complications such as superficial or deep infections, deep venous thrombosis, pulmonary embolism, or wound healing problems.

TKAs in both groups were implanted by two senior orthopedics surgeons (one is the co-author CHY) who have been performing both designs (MP and PS prostheses) for more than 5 years. Standard surgical techniques included a longitudinal skin incision, medial parapatellar approach, subperiosteal dissection over the medial tibial plateau, and resurfacing of the patella depending on the intraoperative wear pattern. Surgery was undertaken under spinal or combined spinal and epidural anesthesia and lasted for 90 to 120 min. Patients received standard, weight-based doses of preoperative antibiotics, and intravenous tranexamic acid, followed by 2 weeks of venous thromboembolism prophylaxis with aspirin. Inpatient physiotherapy was commenced on post-operative day 0 under the management of physiotherapists in order to achieve full weight bearing on the operated leg. Active, active-assisted, and passive knee mobilization exercises were progressively prescribed. Patients were trained to walk with a frame or a quadripod depending on their progress. Additionally, they were given an exercise pamphlet regarding lower limb stretching and mobilization exercises to perform at home following discharge.

Age-matched asymptomatic controls comprised community senior center attendees. Inclusion criteria for asymptomatic controls included: no signs of stiffness or pain in the knees in the last year; and able to walk unaided both indoors and outdoors. Exclusion criteria for asymptomatic controls followed those of participants with TKA. The study was approved by the Human Subjects Ethics Sub-committee of the University (HSEARS20161110003). All participants provided informed consent before participation.

### Sample size

Since the objective of this study was to assess the feasibility of using body-worn sensors to access balance performance of TKA patients with different prothesis design, a formal sample size calculation was not conducted.

### Self-reported pain, stiffness, and physical functioning

Pain, stiffness and physical functioning of all participants were measured with the Chinese version of the Western Ontario and McMaster Universities Osteoarthritis Index (WOMAC) [24]. This activity-based self-administered questionnaire comprises 24 questions related to knee pain (5 items), knee stiffness (2 items), and physical functions (17 items). WOMAC has demonstrated good test–retest reliability for evaluating the TKA population (intra-class correlation coefficient = 0.82, 0.88, 0.84 for pain, stiffness, and function respectively) [24]. A higher score in each subscale indicates that the respondent has more knee pain, more knee stiffness or poorer physical function.

### Mobility assessments

Mobility was assessed with two performance-based tests: the timed-up-and-go test (TUG) and the 6-min walk test (6MWT). These tests are commonly used to evaluate the functional recovery after TKA [25, 26]. The TUG has an excellent reported inter-rater reliability with an intraclass correlation coefficient of 0.99, and is a functional test of strength, agility, and dynamic balance [27]. Participants were instructed to rise from an armless chair (seat height of 46 cm), walk unaided at a self-selected comfortable pace along a line on the floor for 3 m, and then turn and walk back to the chair and sit down [27]. Participants performed a practice trial, followed by two experimental trials with the faster trial time used for analysis. The 6MWT assesses walking endurance/tolerance and has demonstrated excellent inter-rater reliability (intraclass correlation coefficient of 0.91) [28]. The test was demonstrated and then participants were asked to walk as quickly as possible back and forth along a 20-m hallway for 6 min without running or jogging [29]. Participants were allowed to slow down, to stop, or to rest, if necessary. A standard set of recommended instructions and encouraging statements were used [29]. The total distance covered was recorded for analysis.

### Standing balance and gait stability assessments

**Fig. 1:**
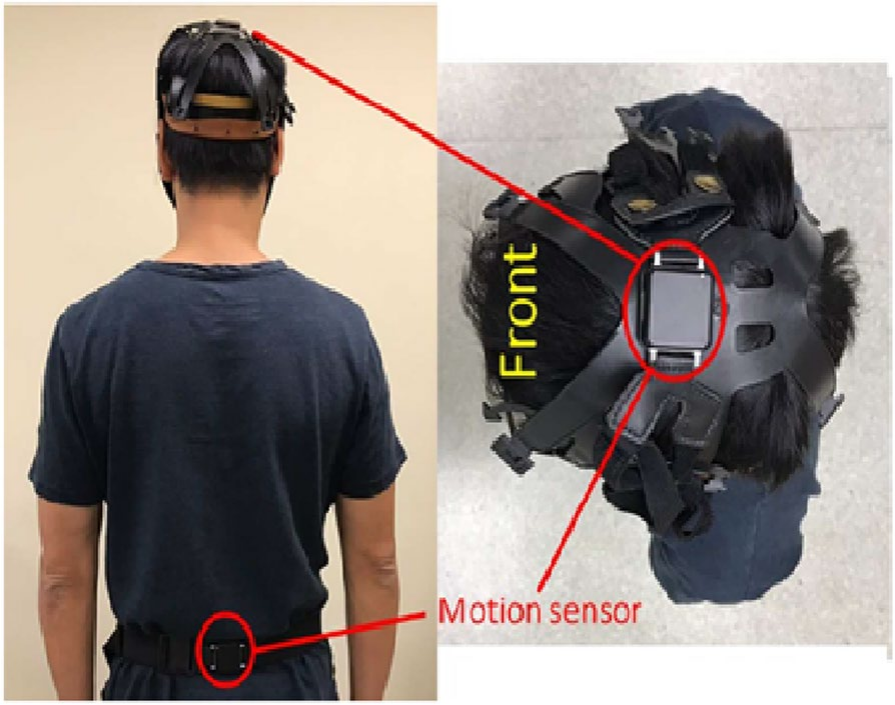
The placements of inertia sensors at the head and waist

Balance parameters during a standing task and during the 6MWT were assessed with two synchronized inertial sensors (Fig. 1). Each sensor contained a tri-axial accelerometer and a tri-axial gyroscope (Opal, APDM Inc, Portland, OR, USA; sampling frequency 128 Hz). The lumbar sensor was firmly strapped onto the participant with a belt approximately at the L5 level (near the body center of mass). The head sensor was attached to a plastic helmet that was secured at the vertex of the participant’s head. The three-dimensional angular velocity and acceleration data from each sensor were collected during both static and dynamic tasks using the built-in software of the APDM Mobility Lab system (Opal, APDM Inc, Portland, OR, USA). The data was then exported as CSV files and processed using a custom-written program (MATLAB, Natick, MA, USA).

**Fig. 2:**
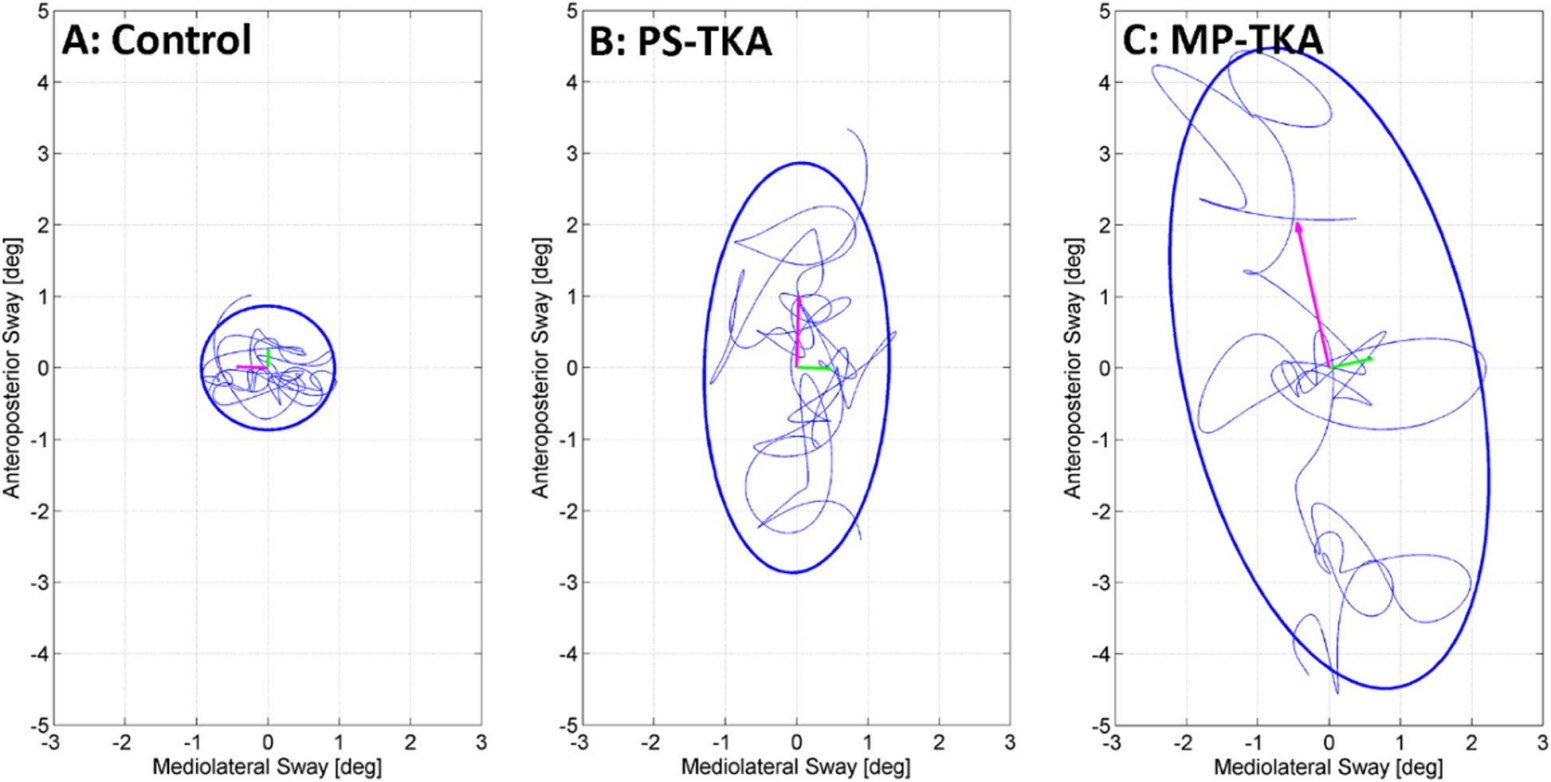
The postural sway of control and patients with two total knee arthroplasty (TKA) prosthesis designs during the near-tandem stance test

Postural sway was assessed using the near-tandem stance test with eyes open. Participants were instructed to fold their arms across the chest and to stand with the heel of front foot (dominant foot: determined by self-reported leg dominance using the question of which leg will be used to shoot a ball on a target regardless of any pain on lower limbs) 2.5 cm anterior and 2.5 cm lateral (marked by a 2.5 cm × 2.5 cm cardboard template) to the great toe of the rear foot on a hard surface for 30 s. The head and lumbar static balance parameters were assessed over the middle 25 s to prevent any movements at the beginning or end of the test affecting the results. Static balance parameters included the 95% range of sway in degrees (ϴ) in the anteroposterior (AP) and mediolateral (ML) directions called pitch and roll, respectively (Eqs. 1 and 2). The root mean square (RMS) of angular velocity was calculated by combining movements about all axes into one parameter (Fig. 2), where fA is the acceleration of the sensor after low-pass filtering with a 4th order bidirectional Butterworth filter at a cut-off frequency of 1 Hz.

1 $${\theta }_{AP(Pitch)}={\mathrm{sin}}^{-1}\frac{{fA}_{AP}}{\sqrt{{fA}_{AP}^{2}+{fA}_{ML}^{2}+{fA}_{VT}^{2}}}$$

2 $${\theta }_{ML(Roll)}={\mathrm{sin}}^{-1}\frac{{fA}_{ML}}{\sqrt{{fA}_{AP}^{2}+{fA}_{ML}^{2}+{fA}_{VT}^{2}}}$$

Note: VT-vertical.

**Fig. 3:**
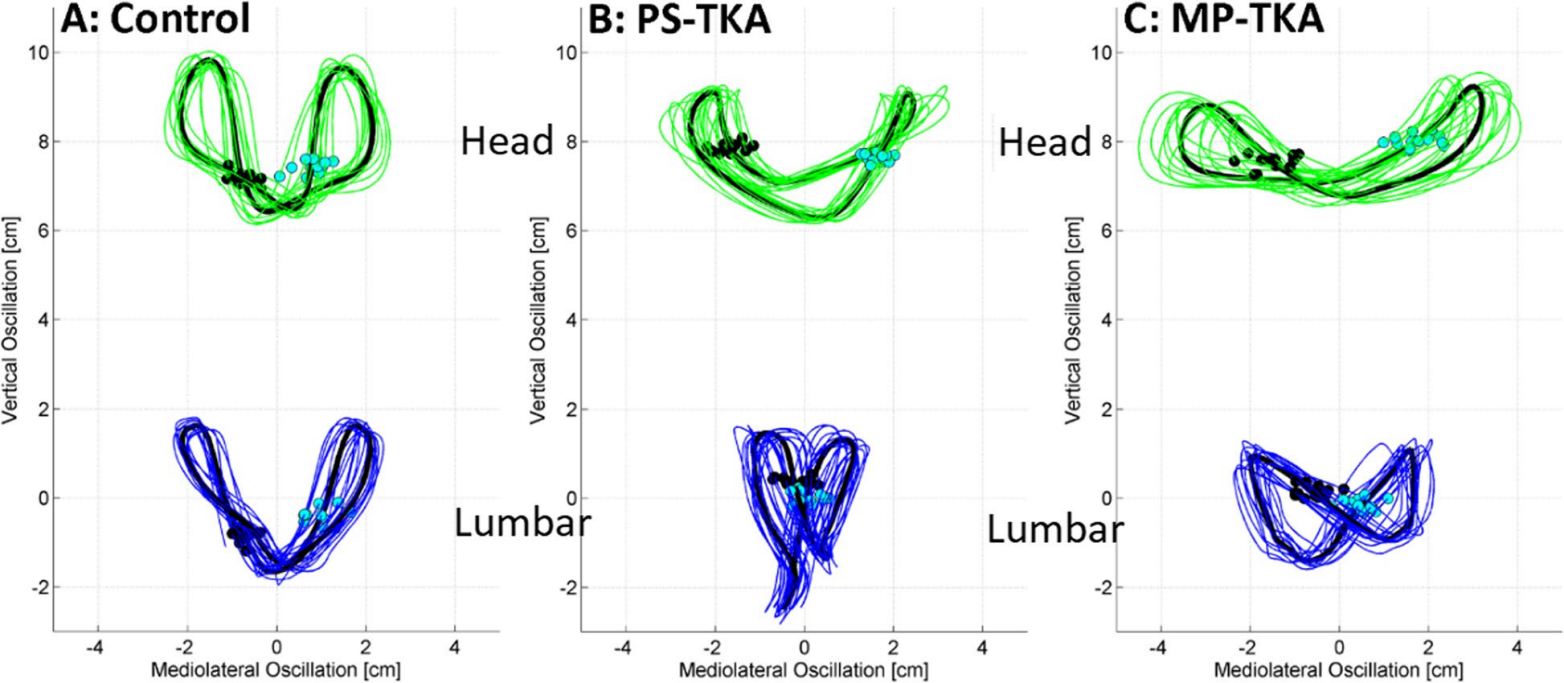
Data collection during 6-min walk test

**Fig. 4:**
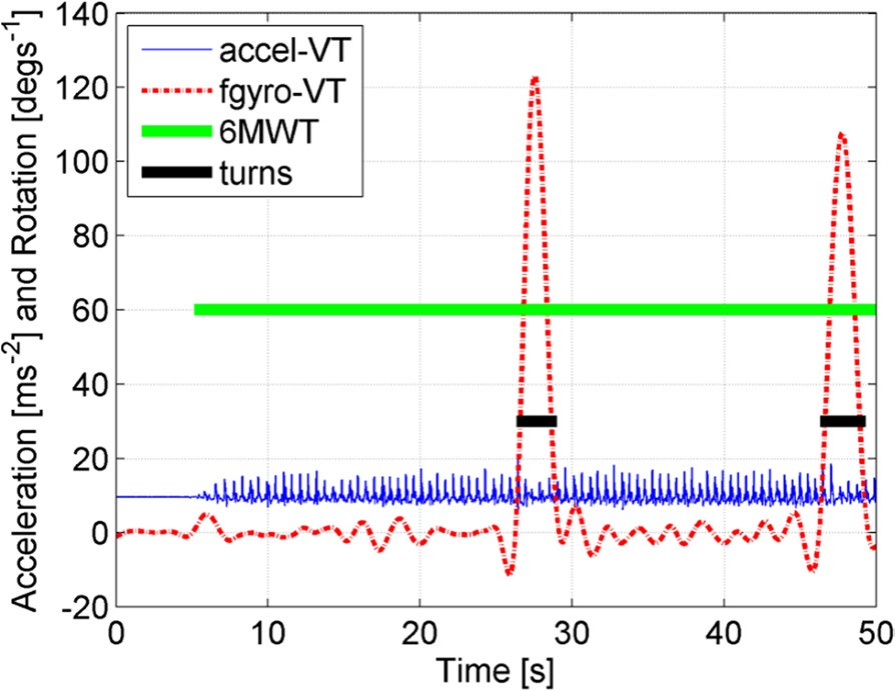
Low-pass filtered was used to identify the real transient gait rotations

Dynamic balance was assessed during the periods of straight line walking in the 6MWT from multiple 20-m laps (Fig. 3). Turns were identified and excluded from the analysis by the gyroscope threshold of 30º/s about the vertical axis. To prevent false identification of transient gait rotations instead of turns the gyroscope data were low-pass filtered using a 4th order bidirectional Butterworth filter with a 0.5 Hz cut-off frequency and a total turn rotation of at least 90º was required (Fig. 4). For each gait parameter, the robust mean was calculated (mean after excluding the best lap and worst lap). Steps during each straight line lap were identified by heel strike acceleration peaks [30]. Cadence was calculated as the number steps per minute. Stride time variability was the standard deviation of consecutive strides (1 stride = 2 steps); greater variability has been associated with increased fall risk [31]. Step time asymmetry was calculated as the absolute difference between left and right step times as a percentage [30]. Relative displacements of the head and the lumbar region were reported as the RMS along the AP, ML, and VT directions based on the sensor data using a validated method [21, 32]; reduced VT displacements correlate with less vigorous gait while increase transverse plane displacements are associated with reduced gait stability [20]. Finally, harmonic ratios (HR) were used as a measure of rhythm/smoothness of head and lumbar accelerations during walking. Lower HRs indicate reduced dynamic balance and are associated with increased fall risk [33, 34].

### Statistical analysis

All statistical analyses were performed using SPSS software (Version 22, IBM Corp., Armonk, NY, USA). Non-parametric tests were used for the analysis as most variables did not meet the requirements for normality as determined by Shapiro-Wilks tests of normality. Demographic variables specific to participants with TKA (e.g., months after operation and the percentage of participants using walking aids outdoor) were compared between the PS-TKA and MP-TKA participants using Mann–Whitney *U* tests (for continuous variables) or chi-square test (for nominal variables). For the remainder of the demographic and clinical variables of interest, Kruskal–Wallis tests (for continuous variables) and chi-square tests (for nominal variables) were used to compare the differences among PS-TKA, MP-TKA, and asymptomatic controls. The significance level was set at 0.05 (2-tailed) and post hoc analysis using Bonferroni adjustments were performed. Effect sizes (*r*) of each observed difference were calculated by dividing the *Z* value by the square root of the total number of participants in that pair of groups [35]. Cohen’s guidelines for *r* suggest that small, medium and large effect sizes are 0.1, 0.3, and 0.5 respectively [36].

## Results

### Participants

**Fig. 5:**
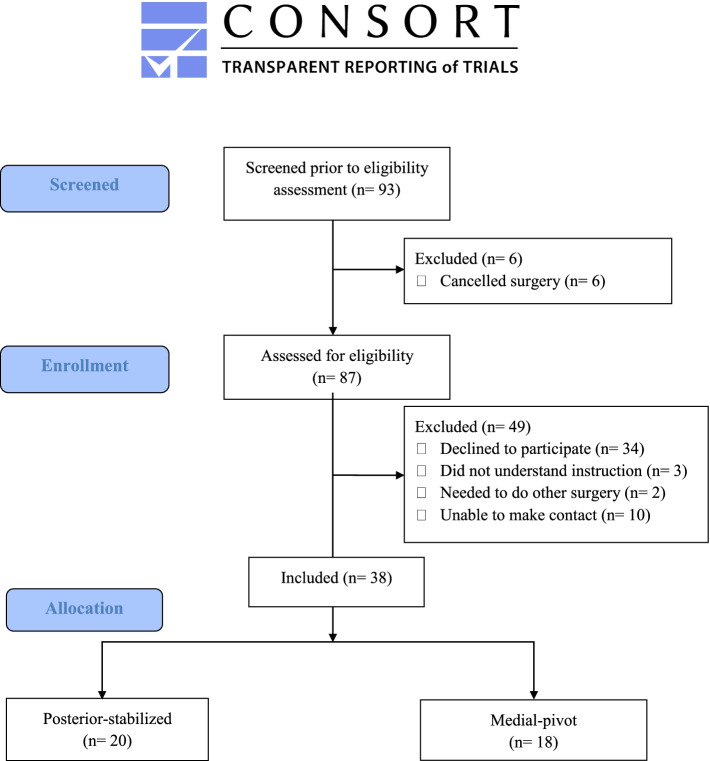
Flow diagram of participants’ recruitment

Ninety-three patients admitted to a local hospital for TKA between December 2017 and February 2019 were screened for eligibility. Twenty PS-TKA and 18 MP-TKA met the inclusion criteria and agreed to participate in the study (Fig. 5). Nineteen age-matched asymptomatic controls were recruited from a community center. Demographic data are shown in Table 1. There were no significant age and gender differences among the groups. The two TKA groups showed no significant differences in post-surgery duration, number of falls or trips within 1 year before surgery, number of falls or trips before and after operation, and the proportions using an assistive device outdoors. The controls took significantly fewer medications and had significantly lower BMIs, whereas the two TKA groups did not differ significantly with respect to these parameters. There were no significant differences in the number of falls or trips in the previous 12 months among the three groups.

**Table 1:** Participant characteristics [median (interquartile range)]

	PS-TKA (*n* = 20)	MP-TKA (*n* = 18)	Controls (*n* = 19)	*P* value
Age (years)	71.00 (65.50 to 74.50)	75.00 (60.75 to 81.00)	68.00 (61.05 to 71.00)	0.191
Weight (kg)	68.25 (63.75 to 73.05)	67.50 (64.10 to 75.28)	57.00 (50.15 to 63.15)	0.010*
Height (m)	1.54 (1.49 to 1.58)	1.53 (1.48 to 1.61)	1.55 (1.49 to 1.60)	0.033*
BMI (kg/m^2^)	27.93 (26.94 to 30.35) ^†^	28.42 (26.68 to 29.75) ^†^	22.83 (21.36 to 24.93)	< 0.001^*^
Female:male	19:1	13:5	16:3	0.173
Months after operation (months)	13.00 (10.00 to 14.00)	12.00 (10.00 to 14.00)	NA	0.511
At least one medication (% yes)	70.00%^†^	72.00%^†^	16.00%	0.007^*^
Falls 1 year before operation (% yes)	30.00%	11.00%	NA	0.154
Trips 1 year before operation (% yes)	35.00%	28.00%	NA	0.632
Falls after operation/in the last 12 months (% yes)	10.00%	6.00%	10.00%	0.825
Trips after operation/ in the last 12 months (% yes)	40.00%	28.00%	37.00%	0.443
Pre- vs post-operation falls	6 vs 2	2 vs 1	NA	NS
Pre- vs post-operation trips	7 vs 8	5 vs 5	NA	NS
Use of walking aids outdoor (% yes)	55.00%	62.00%	0.00%	0.343

*BMI* body mass index, *NS* not significant, *NA* not applicable

^*^Denotes significant difference at *p* < 0.05 using Kruskal–Wallis test

^†^Denotes significant difference from control at *p* < 0.05/3 using Mann–Whitney *U* test with Bonferroni adjustment

### Pain, stiffness, and physical functioning

No significant differences were detected in self-reported knee pain, knee stiffness and physical activity limitations between the two TKA groups. However, both PS-TKA and MP-TKA participants perceived significantly more knee pain, knee stiffness, and physical activity limitations than the asymptomatic controls (Table 2).

**Table 2:** WOMAC scores and mobility test performances [median (interquartile range)]

	PS-TKA (*n* = 20)	MP-TKA (*n* = 18)	Controls (*n* = 19)	K-W	PS-TKA vs MP-TKA		PS-TKA vs Controls		MP-TKA vs Controls	
				*p* value	*p* value	ES	*p* value	ES	*p* value	ES
WOMAC–pain (out of 21)	2.37 (1.50 to 4.59)	2.35 (0.35 to 4.37)	0.00 (0.00 to 0.20)	< 0.001^*^	0.632	− 0.05	** < 0.001**^**†**^	− **0.54**	**0.002**^**†**^	− **0.42**
WOMAC–stiffness (out of 8)	1.74 (1.07 to 4.03)	1.06 (0.61 to 3.01)	0.00 (0.00 to 0.10)	** < 0.001**^**†**^	0.360	− 0.30	** < 0.001**^**†**^	− **0.59**	**0.001**^**†**^	− **0.54**
WOMAC–physical function (out of 71)	14.90 (3.88 to 24.90)	7.40 (4.76 to 13.72)	0.40 (0.00 to 1.60)	< 0.001^*^	0.560	− 0.20	** < 0.001**^**†**^	− **0.62**	** < 0.001**^**†**^	− **0.55**
WOMAC–total score	19.12 (8.53 to 30.20)	10.44 (7.10 to 17.49)	0.80 (0.00 to 2.50)	< 0.001^*^	0.577	− 0.20	** < 0.001**^**†**^	− **0.60**	** < 0.001**^**†**^	− **0.56**
6MWT distance (m)	330.26 (284.96 to 356.70)	321.94 (254.62 to 372.96)	382.42 (359.33 to 459.40)	< 0.001^*^	0.876	− 0.02	** < 0.001**^**†**^	− **0.50**	** < 0.001**^**†**^	− **0.52**
TUG time (s)	11.87 (10.06 to 15.40)	13.93 (10.97 to 15.18)	11.62 (10.00 to 12.94)	0.011^*^	0.739	− 0.04	0.055	− 0.09	0.019	− 0.52

*ES* effect size, *K-W* Kruskal–Wallis test, *MP-TKA* medial-pivot total knee arthroplasty, *PS-TKA* posterior-stabilized total knee arthroplasty, *TUG* timed-up-and-go test, *WOMAC* Western Ontario and McMaster Universities Osteoarthritis Index, *6MWT* six-minute walk test

^*^Denotes significant difference at *p* < 0.05 using Kruskal–Wallis test

^†^Denotes significant difference from control at *p* < 0.05/3 using Mann–Whitney *U* test with Bonferroni adjustment

### Mobility assessments

No significant difference in 6MWT distance was noted between the two TKA groups. Compared to the asymptomatic controls, both TKA groups had significantly shorter 6MWT walking distances. There was a trend indicating the MP-TKA group had slower TUG times than the control group (Table 2).

### Postural sway in the near-tandem stance test

In the near-tandem stance test, significant differences in sway in the AP direction assessed the level of the head and lumbar regions were observed among the three groups (Table 3). Post hoc analyses showed that the MP-TKA participants has greater head and lumbar AP sway than the asymptomatic controls, but that these sway measures in both regions did not differ between the two TKA groups.

**Table 3:** Postural way in the near-tandem quiet standing test and gait parameters in the 6-min walk test [median (interquartile range)]

	PS-TKA (*n* = 20)	MP-TKA (*n* = 18)	Controls (*n* = 19)	K-W	PS-TKA vs MP-TKA		PS-TKA vs Controls		MP-TKA vs Controls	
				*p* value	*p* value	ES	*p* value	ES	*p* value	ES
***The near tandem quiet standing test***										
**Lumbar**										
AP sway range (°)	1.36 (1.03 to 2.16)	1.69 (1.16 to 2.33)	1.26 (0.81 to 1.38)	0.041^*^	0.287	− 0.13	0.117	− 0.32	**0.011 **^**†**^	** − 0.49**
ML sway range (°)	1.74 (1.15 to 2.24)	1.53 (1.03 to 2.45)	2.15 (1.15 to 2.67)	0.953	0.717	0.06	0.443	− 0.22	0.822	− 0.15
RMS angular velocity (°/s)	1.69 (1.29 to 1.94)	1.69 (1.43 to 2.36)	1.33 (1.10 to 2.38)	0.784	0.601	− 0.08	0.255	− 0.01	0.301	− 0.05
**Head**										
AP sway range (°)	2.65 (2.05 to 3.96)	3.55 (2.41 to 4.52)	2.26 (1.69 to 2.96)	0.023^*^	0.234	− 0.28	0.100	− 0.28	**0.006 **^**†**^	** − 0.35**
ML sway range (°)	3.59 (2.34 to 4.73)	4.20 (2.10 to 7.51)	3.70 (2.32 to 5.06)	0.219	0.355	− 0.15	0.717	− 0.01	0.796	− 0.01
RMS angular velocity (°/s)	2.79 (1.85 to 3.28)	3.27 (2.25 to 3.93)	2.41 (1.98 to 4.41)	0.314	0.212	− 0.20	0.959	− 0.03	0.301	− 0.08
***6-min walk test***										
Cadence (steps/min)	109.97 (102.70 to 113.96)	110.13 (102.90 to 114.91)	113.87 (113.00 to 119.64)	0.004^*^	0.585	− 0.02	**0.006**^**†**^	** − 0.31**	0.033	− 0.40
Stride time variability (ms)	23.86 (19.19 to 31.64)	23.09 (19.00 to 30.89)	16.46 (12.58 to 19.28)	> 0.001^*^	0.984	− 0.03	**0.001 **^**†**^	** − 0.50**	**0.001 **^**†**^	** − 0.52**
Step time asymmetry (%)	3.09 (1.34 to 7.88)	3.80 (1.85 to 5.74)	1.34 (0.80 to 2.76)	0.005^*^	0.516	− 0.12	0.046	− 0.04	**0.007 **^**†**^	** − 0.40**
Lumbar displacement (cm)										
RMS AP	0.94 (0.80 to 1.10)	0.88 (0.66 to 0.98)	1.00 (0.92 to 1.13)	0.138	0.235	− 0.19	0.737	− 0.08	0.098	− 0.27
RMS ML	1.41 (1.28 to 1.59)	1.49 (1.27 to 2.05)	1.08 (0.74 to 1.36)	0.002^*^	0.351	− 0.01	0.035	− 0.40	**0.002 **^**†**^	** − 0.49**
RMS VT	0.99 (0.91 to 1.18)	0.91 (0.83 to 1.21)	1.30 (1.07 to 1.62)	0.001^*^	0.482	− 0.11	**0.009 **^**†**^	− 0.40	**0.001 **^**†**^	** − 0.50**
Head displacement (cm)										
RMS AP	0.32 (0.27 to 0.40)	0.35 (0.27 to 0.47)	0.31 (0.22 to 0.37)	0.640	0.658	− 0.07	0.698	− 0.09	0.191	− 0.16
RMS ML	2.64 (1.98 to 3.06)	2.35 (1.80 to 2.87)	1.45 (1.05 to 1.83)	< 0.001^*^	0.875	− 0.08	** < 0.001**^**†**^	** − 0.54**	** < 0.001 **^**†**^	** − 0.55**
RMS VT	0.87 (0.751 to 1.02)	0.89 (0.73 to 1.12)	1.15 (1.05 to 1.53)	< 0.001^*^	0.699	− 0.06	**0.001**^**†**^	** − 0.45**	**0.003**^**†**^	** − 0.41**
Lumbar harmonic ratio										
AP	1.83 (1.63 to 2.05)	1.67 (1.53 to 1.79)	1.95 (1.75 to 2.19)	0.010^*^	0.273	− 0.34	0.542	− 0.33	**0.007 **^**†**^	** − 0.38**
ML	0.76 (0.66 to 0.88)	0.79 (0.73 to 0.96)	0.83 (0.77 to 1.00)	0.020^*^	0.942	− 0.04	0.109	− 0.31	0.017	− 0.42
VT	1.88 (1.62 to 1.97)	1.67 (1.44 to 1.85)	1.90 (1.80 to 2.11)	0.011^*^	0.633	− 0.15	0.241	− 0.21	**0.008 **^**†**^	** − 0.44**
Head harmonic ratio										
AP	1.41 (1.27 to 1.48)	1.41 (1.28 to 1.47)	1.56 (1.47 to 1.79)	< 0.001^*^	0.903	− 0.07	**0.001 **^**†**^	** − 0.37**	** < 0.001**^**†**^	** − 0.53**
ML	0.87 (0.80 to 1.02)	0.82 (0.80 to 0.92)	0.89 (0.76 to 0.95)	0.015^*^	0.965	− 0.14	0.034	− 0.48	**0.009 **^**†**^	** − 0.44**
VT	1.99 (1.77 to 2.15)	1.91 (1.72 to 2.15)	2.14 (2.01 to 2.44)	0.003^*^	0.699	− 0.06	0.019	− 0.32	**0.005 **^**†**^	** − 0.40**

*AP* anteroposterior, *ES* effect size, *K-W* Kruskal–Wallis test, *ML* mediolateral, *MP-TKA* medial pivot total knee arthroplasty, *PS-TKA* posterior-stabilized total knee arthroplasty, *RMS* root mean square, *VT* vertical

^*^Denotes significant difference at *p* < 0.05 using Kruskal–Wallis test

^†^Denotes significant difference from control at *p* < 0.05/3 using Mann–Whitney *U* test with Bonferroni adjustment

### Gait speed and stability

Signicant differences were observed for cadence, stride time variabilty and step time asymmetry among the three groups (Table 3). Post hoc analyses revealed that both MP-TKA and PS-TKA participants demonstrated significantly greater stride time variability and step time asymmetry than asymptomatic controls, while PS-TKA participants had significantly reduced cadence and greater stride time variability than asymptomatic controls. The two TKA groups did not differ significantly in any gait parameters.

Regarding oscillatory displacements during the 6MWT, significant differences were observed in the ML and VT directions (Fig. 3, Table 3). Compared to asymptomatic controls, the MP-TKA participants had significantly greater ML oscillatory displacements and significantly smaller VT oscillatory displacements assessed at both the head and lumbar levels. For the PS-TKA participants, these differences were only detectable at the level of the head (Table 3).

The three groups demonstrated significantly different HRs all the three axes at both the head and lumbar levels during the 6MWT (Table 3). Post hoc tests revealed that the MP-TKA participants had significantly lower head and lumbar HRs than asymptomatic controls in 5 of the 6 parameters, while the PS-TKA participants only had significantly lower head HRs in AP axis than the asymptomatic controls. No significant difference in HRs were evident between the two TKA groups.

### Feasbility of using wearable devices for data collection

It was feasible to collect mobility and balance data using body-worn devices in a clinical setting. The average data collection duration was 20 min (including breaks between tests). All participants did not experience discomfort in wearing the devices. However, a few participants with a small head circumference reported that the plastic helmet (used for holding the head sensor) was relatively loose. Therefore, they tried to keep the helmet in place to avoid it from falling off the head during 6MWT. To minimize this problem, the adjustable strap lock of the plastic helmet was modified to secure the helmet on patients with smaller head sizes.

### Feasibility of the present protocol for the future trial

This study provided a justification to conduct an RCT to test the hypothesis regarding potential differences in the impacts of two TKA designs on the balance/mobility of patients 1 or 2 years after TKA, which may guide the selection of TKA prostheses and new prosthesis development.

## Discussion

This pilot study found that there were no significant differences in pain, balance, and mobility outcomes between the two TKA groups at 1-year post-surgery. Both TKA groups reported more pain, demonstrated inferior physical performances (e.g., shorter 6MWT distances), increased postural sway in a challenging stance position and poorer gait. Specifically, both TKA groups had increased stride-time-variability and reduced harmonic ratios, which together indicate gait instability [37]. Importantly, our study showed that it was feasible to use body-worn motion sensors in a clinical setting to compare differences in balance, gait stability, and physical performance of patients with PS-TKA and MP-TKA, and age-matched asymptomatic controls. Since no adverse effects were noted in using this fast and non-invasive assessment method, wearable motion sensors are suitable for evaluating balance and physical performance in future clinical trials.

The TKA designs may play a role in dynamic trunk control during walking. Recent research has shown that the knee flexes from 0^◦^ to 45^◦^ during stance phase, which involves a lateral-pivot kinematic pattern of the knee (from heel strike to flat foot). This is followed by the medial-pivot pattern in later flexion range (from flat foot to push off) [38, 39]. The medial-pivot kinematics adopted in the MP-TKA may limit the natural lateral-pivot pattern in the early flexion during walking due to the lack of lateral constraint combined with a single radius of curvature femoral component design (instead of a traditional multi-radius design) and more normal tensioning of the collateral ligaments [40]. Conversely, since the PS-TKA prosthesis allows mediolateral posterior translation during flexion [5], the lateral-pivot pattern is facilitated by PS-TKA. Hence, PS-TKA patients may be expected to have better step time symmetry and better control of lateral oscillations during gait as compared to MP-TKA patients.

While there was a trend showing MP-TKA participants may have less WOMAC stiffness (effect size *r* = 0.34) and better physical functioning (effect size *r* = 0.20) than PS-TKA participants, no significant difference was detected in the WOMAC pain, stiffness, and functioning outcomes between 2 TKA groups. The lack of significant differences in these WOMAC domains between the PS-TKA and MP-TKA concurs with previous research that has reported no significant differences in self-reported clinical outcomes between PS-TKA and MP-TKA patients 5 to 6 years after TKA [16, 41]. Warth et al. observed that the presence of an intraoperative medial-pivot kinematic pattern of TKA implants was not necessarily associated with better subjective outcomes at 1 year post-surgery [42].

The poorer self-reported functional status of both PS-TKA and MP-TKA participants as compared to asymptomatic controls in the current study is consistent with prior studies [13, 43], although Yoshida et al. found that self-reported functional status in TKA patients was equivalent to asymptomatic controls at 12 months post-surgery [26].

The TKA groups showed comparable TUG performances, but poorer 6MWT performances compared with asymptomatic controls. The shorter 6MWT distances in TKA groups is in line with two previous studies conducted in people with unspecified TKA of similar ages [25, 43]. However, Yoshida et al. found no significant differences in TUG and 6MWT results between post-TKA participants and asymptomatic controls at 1 year after TKA [26]. Interestingly, the average performance of TKA participants in the abovementioned studies were better than that of our TKA participants and asymptomatic controls. These differences might be attributed to the fact that our TKA participants did not undergo any structured post-operative rehabilitation programs, resulting in lower physical activity levels [44]. Future prospective studies should investigate whether post-operative rehabilitation could help close these substantial gaps.

With respect to standing balance, AP sway measured at the head and lumbar regions differed between the two TKA groups and the asymptomatic controls, whereas no such differences were evident for ML sway. Previous research has reported that participants place approximately 70% of their body weight on the rear foot during the tandem stance [45], while the AP rhythmic weight shifting plays a functional role in maintaining lateral stability [45]. Increased AP sway in MP-TKA patients may indicate that they are less capable in detecting or controlling their sway in a challenging balancing stance, or they need to transfer weight between the two feet to maintain their lateral stability. The trend indicated that MP-TKA participants may have poorer standing balance than PS-TKA counterparts (Fig. 2).

There were no significant differences in stride-to-stride oscillations and HR gait parameters between the two TKA groups (Table 3), but cadence was decreased, VT displacement was reduced, and ML displacement, stride time variability, and step time asymmetry were increased in both TKA groups compared with the asymptomatic controls (Fig. 3). The two TKA groups demonstrated reduced HRs, indicating less smooth and more unstable walking patterns [44, 45]. Collectively, these results indicate both TKA groups had suboptimal dynamic stability even at 1 year after surgery. Further, a lower AP HR of lumber was observed in MP-TKA patients. It is postulated that MP-TKA participants may have a lower stability when walking resulting in increased compensatory movement in lumber than PS-TKA counterparts (effect size *r* = 0.34).

While our results supported the use of wearable motion sensors to evaluate balance of TKA patients in a clinical setting, the participants’ inclusion criteria and assessment tasks should be modified in the future planned RCT. First, since many patients with bilateral severe knee osteoarthritis in Hong Kong may need to undergo second TKA in another knee between 12 and 18 months after receiving the first unilateral TKA, they cannot be followed up at 2 years post-operatively. The planned trial should recruit patients undergoing second TKA that is identical to their first TKA prosthesis in another knee so that they can be followed up for a long period. Second, some participants expressed difficulty in using the continuous visual analog scales (scores ranging from 0 to 100 mm) of WOMAC. Since older adults desired fewer choice options [46], items rated on a 5-point Likert scale may be a good alternative. Third, level ground walking may be insensitive to detect differences in functional performance between the two TKA groups because the medial-pivot pattern only occurs at knee flexion beyond 45° in natural knee kinematics [38]. Future research should include well-established balance tests (e.g., Brief-BESTest) [47] and additional functional tasks (e.g., 30-s chair-stand test, a stair-climb test) [46] to detect subtle differences in functional performance/balance control between PS-TKA and MP-TKA patients.

The limitations of this study include: first, mild knee osteoarthritis in the non-operated knee of some TKA participants might have affected their pain, balance and mobility. Second, unmeasured physical factors (e.g., impaired vision, proprioception and muscle strength) might have influenced our findings although people with diagnosed visual and proprioception impairments were excluded. Third, multiple outcomes were measured given the exploratory nature of this study; factors that might have resulted in both type 1 and type 2 errors. Further, the sample size was relatively small and thus might have been underpowered with respect to some outcome measures. However, both self-reported and objective measures consistently indicated that TKA participants demonstrated significantly poorer balance and mobility than age-matched asymptomatic controls. The sample size for a potential definitive RCT was calculated based on the difference in the WOMAC total score. It was estimated that 36 participants per group was needed to detect the observed difference with 80% power, a significant level of 0.05 in the future definitive RCT. Fourth, since participants were not assessed pre-operatively nor randomized into different TKA groups before their surgeries, it was impossible to rule out pre-existing differences in the health condition or functional abilities between the two TKA groups. However, this case–control study evaluated the feasibility of using wearable sensors to compare the static and dynamic balance of patients with different TKA protheses at 1 year after surgery. It helped refine the objective balance assessment procedures for future studies (e.g., balance assessments in patients with different TKA prosthesis designs, or post-TKA rehabilitation) [48].

Collectively, this cross-sectional pilot study substantiated that it was feasible and safe to use non-invasive wearable motion sensors in a clinical setting to detect static/dynamic balance and physical performance of patients with different TKA designs. Although our preliminary results showed no significant differences in pain intensity, balance, and functional performance of patients with MP-TKA and PS-TKA 1 year after surgery, these patients had significantly poorer clinical outcomes than age-matched asymptomatic controls. Given that our findings were preliminary and based on small sample size, future RCTs should adopt the current assessment protocol alongside the discussed modifications to evaluate the effects of different TKA prostheses on functional outcomes of TKA patients after two or more years post-surgery.

## Supplementary Information

**Additional file 1: Supplementary file 1.** CONSORT 2010 checklist of information to include when reporting a pilot or feasibility randomized trial in a journal or conference abstract 

## Data Availability

The datasets generated and/or analyzed during the current study are not publicly available due to personal data confidentiality policy but are available from the corresponding author on reasonable request.

## Abbreviations

6MWT: Six-minute walk test
AP: Anteroposterior
BMI: Body mass index
HR: Harmonic ratios
ML: Mediolateral
MP: Medial-pivot
PS: Posterior-stabilized
RMS: Root mean square
TKA: Total knee arthroplasty
TUG: Timed-up-and-go
VT: Vertical
WOMAC: Western Ontario and McMaster Universities Osteoarthritis Index
